# Effectiveness of osseodensification in enhancing implant stability and ridge expansion in esthetic zone: a randomized controlled clinical trial

**DOI:** 10.1007/s10266-025-01284-1

**Published:** 2026-01-06

**Authors:** Mohamed Ahmed Hammouda, Mona Elsayed Lotfy, Souzy Kamal Anwar

**Affiliations:** Oral Medicine, Periodontology, Oral Diagnosis, and Oral Radiology Department, Faculty of Dentistry, Alexandria University, Champollion St., Azarita, Alexandria, 21521 Egypt

**Keywords:** Bone expanders, Densah burs, Dental implant, Osseodensification, Osstell

## Abstract

The anterior or esthetic region frequently exhibits compromised bone volume and quality, posing challenges for implant placement. Attaining optimal implant stability while preserving or augmenting ridge dimensions is essential for both functional success and long-term esthetic outcomes. Osseodensification, a biomechanical site preparation technique, compacts rather than removes bone, potentially enhancing stability and facilitating ridge expansion. Our research aimed to compare osseodensification with conventional manual bone expanders in enhancing implant stability and ridge expansion in the esthetic zone. A randomized controlled clinical trial was conducted on eighteen patients missing a maxillary anterior tooth with moderate horizontal ridge deficiency (3–4 mm width, ≥ 10 mm height). Participants were equally allocated into two groups: Group I (osseodensification), where sites were prepared using Densah burs in densifying mode, and Group II (manual expanders), prepared with Microdent expanders. Implants were assessed for primary stability at placement and secondary stability at 16 weeks using resonance frequency analysis. Cone-beam computed tomography was performed at baseline and 16 weeks to evaluate ridge expansion, bone density, and marginal bone loss. Both groups demonstrated significant intra-group increases in ridge width and bone density. No significant differences were noted between groups in ridge expansion or marginal bone loss. However, osseodensification yielded significantly higher secondary stability values compared with expanders. Within the study’s limitations, osseodensification enabled safe ridge expansion and simultaneous implant placement in the esthetic zone, providing superior stability without observed complications. It represents a predictable, minimally invasive technique that supports successful and esthetically favorable implant outcomes.

*Trial registration* NCT06667778 retrospectively on 31/10/2024, Clinicaltrial.gov.

## Introduction

In dental practice, using dental implants to replace a lost natural tooth has become standard practice [1]. However, the anterior maxilla presents unique challenges due to esthetic demands and bone quality. Studies have reported survival rates of approximately 95.7% in this region over a follow-up period of 3 years [2]. One important factor influencing implant stability is thought to be bone density [3]. While bone strength is directly connected to mineral density and collagen integrity, the quality and quantity of histological structure at the implant interface are highly correlated with bone density at the implant site [4].

For implant durability and long-term clinical success, bone volume must be maintained throughout osteotomy preparation. As defined as “a direct structural and functional connection between organized, living bone, and the surface of a load-bearing implant,” osseointegration is a critical factor in implant success [5, 6]. For osseointegration to be successful and for implant therapy to be effective over time, primary implant stability must be guaranteed [7]. During surgical insertion, the implant surface and the bone walls at the osteotomic location mechanically interact to produce this stability. As a result, a number of factors unique to each patient, surgical technique, and implant influence the attainment of primary implant stability [8].

To create a cylindrical hole that can hold an implant fixture, osteotomy preparation using traditional drilling techniques involves cutting and extracting bone. Over the past few decades, a variety of surgical techniques have been developed to improve the local bone conditions at implant recipient sites in order to conserve the current bone volume and increase its density, especially in cases where bone density is low [9]. It has been proposed that in low-density bone, omitting the bone tapping step could improve the main implant durability. On the other hand, others recommended to prepare the implant site with an inadequate size [10, 11].

In order to decrease bone cutting and improve primary implant stability in cases of low-quality bone, Degidi et al. [12] reported that a 10% undersized osteotomic implant site preparation relative to the implant diameter. The crestal part of the implant site follows a standard protocol in the stepped osteotomy technique, while the apical part undergoes under preparation [13].

The bone condensation technique with osteotomes is another surgical method intended to increase localized bone density. Using the mechanical action of cylindrical steel instruments that run along the osteotomes’ walls, this technique compresses the bone [14]. This technique creates a tiny pilot hole, after which the bone tissue is compressed laterally and apically using a spreader or implant-shaped instrument [15]. By compressing and manipulating the peri-implant bone, an osteotome can help improve primary stability without causing bone loss [16].

However, according to some authors, this treatment may result in debris-filled trabecular fractures that hinder the osseointegration process [15]. Moreover, some authors have made assertions about the potential for bone loss when utilizing an osteotome to increase bone width. This may be because stress concentrates at the crest when the osteotomy’s diameter increases [17].

In the past, osteotomes were frequently used for treatments involving bone expansion. Because osteotomes are method sensitive and might result in greater stresses that may induce blood flow disruption, bone marrow space displacement, or both, technique is crucial. Additionally, this may compromise the physical integrity of the collagen fibers and cause microscopic damage to trabecular bone. The mouth’s limited opening further limits its use in the posterior maxilla [18].

Recent years have seen the introduction of an implant bed preparation method based on the concept of osseodensification (OD) drilling, which protects bone and eliminates the need for site extraction [19].

Osseodensification is one technique for biomechanically preparing a location. It generates minimum plastic deformation because of its non-extraction site preparation procedure, which helps to conserve bone and increase implant stability [20].

Through the use of a multi-fluted densifying bur technology, this approach uses a new, highly controllable, rapid, and effective procedure with minimal heat elevation to construct and extend a pilot hole without removing a substantial quantity of bone tissue [21]. Because of the taper design, which allows the surgeon to adjust irrigation and pressure and offers special real-time haptic feedback, the densifying bur is simple to use for any experienced implant surgeon [20].

Recent literature has highlighted the importance of understanding the biomechanical stresses generated within bone during OD. These stresses, resulting from the compaction and plastic deformation of trabecular bone, may influence bone cell activity and the subsequent tissue response. Controlled stress levels can stimulate bone remodeling and enhance osseointegration, whereas excessive stress may risk microfracture formation and delayed healing. Such biomechanical insight offers a broader understanding of how densification-induced strain affects peri-implant bone behavior and clinical outcomes [22].

Therefore, we were interested to investigate the effectiveness of using Densah burs in comparison to conventional manual bone expanders in enhancing implant stability and ridge expansion.

The null hypothesis was that there would be no significant difference in implant stability between the two methodologies.

## Materials and methods

### Study design and setting

A two-arm parallel randomized controlled clinical trial was conducted on a convenience sample of eighteen eligible patients selected from those attending the clinic of the Department of Oral Medicine, Periodontology, Diagnosis, and Oral Radiology at the Faculty of Dentistry, Alexandria University, Egypt.

### Ethical approval

The current study received ethical approval from the Research Ethics Committee of the Faculty of Dentistry, Alexandria University, Egypt (IRB No.: 00010556-IORG0008839) and was assigned the serial number 0105-12/2019. It was also conducted in full compliance with the principles outlined in the modified Declaration of Helsinki for human clinical research (2013) [23] and adhered to the CONSORT guidelines (2010) [24] for reporting randomized clinical trials. Prior to enrollment, the study protocol was thoroughly explained to all participants and written informed consent was obtained. Furthermore, the study was registered in the U.S. National Institutes of Health Clinical Trials Registry under the identifier NCT06667778.

### Eligibility criteria

Participants included in the study were individuals aged from 20 to 45 years who demonstrated good oral hygiene, were free from systemic conditions that could impair the healing process, and had a missing maxillary anterior tooth with a moderate horizontal ridge defect, defined as an alveolar ridge width of 3–4 mm and a minimum height of 10 mm [25, 26]. Additionally, participants were required to have sufficient inter-occlusal space for fixed prosthesis placement and adequate keratinized mucosa [27]. We excluded individuals who were heavy smokers (defined as consuming more than 10 cigarettes per day), had uncontrolled diabetes, were immunosuppressed or undergoing radiotherapy or chemotherapy, had a history of alcohol or drug abuse, were diagnosed with osteoporosis, or were pregnant or lactating [28, 29].

### Sample size estimation

Sample size estimation was based on a confidence level = 95%, study power of 80% and bone-to-implant contact (BIC) as an indicator of implant primary stability [30]. The mean percentage of BIC = 31.4% in the conventional osteotome technique [31]. BIC in case of osseodensification using counter-wise Densah burs = 39% with pooled standard deviation = 5 [32]. The minimum required sample size was determined using the G*Power 3.0.10 sample size calculator, resulting in eight participants per group. To account for potential loss to follow-up, this number was increased to nine per group. Thus, the total sample size was calculated as number of groups × participants per group = 2 × 9 = 1 [33, 34].

### Study participants’ grouping and randomization

18 eligible participants were randomly divided into two equal groups. Group I (study group) consisted of nine patients with nine implant sites, which were prepared using osseodensification with Densah burs following the Versah protocol, running in a non-cutting counterclockwise (CCW) direction (Densifying Mode). Group II (control group) also included nine patients, each with an edentulous maxillary anterior region, where nine implant sites were prepared using conventional manual bone expanders (Microdent) according to the manufacturer’s guidelines.

Simple randomization was used to assign participants who met the eligibility criteria. A computer-generated list of random numbers was created (https://www.sealedenvelope.com/simple-randomiser/v1/lists), and each allocation was represented by a code. The codes were sealed in sequentially numbered opaque envelopes, which were opened at the time of the intervention.

### Intervention

For both groups, the pre-surgical phase involved several key steps. First, a thorough patient history was taken, including personal, medical, and dental histories. Clinical and radiographic examinations were performed, including both extraoral and intraoral assessments, along with a panoramic X-ray and CBCT to evaluate ridge dimensions. We then performed phase I therapy and instructed patients to maintain oral hygiene measures. Finally, a conventional acrylic surgical stent was fabricated based on diagnostic wax-ups to guide the desired restorative outcome.

**Fig. 1:**
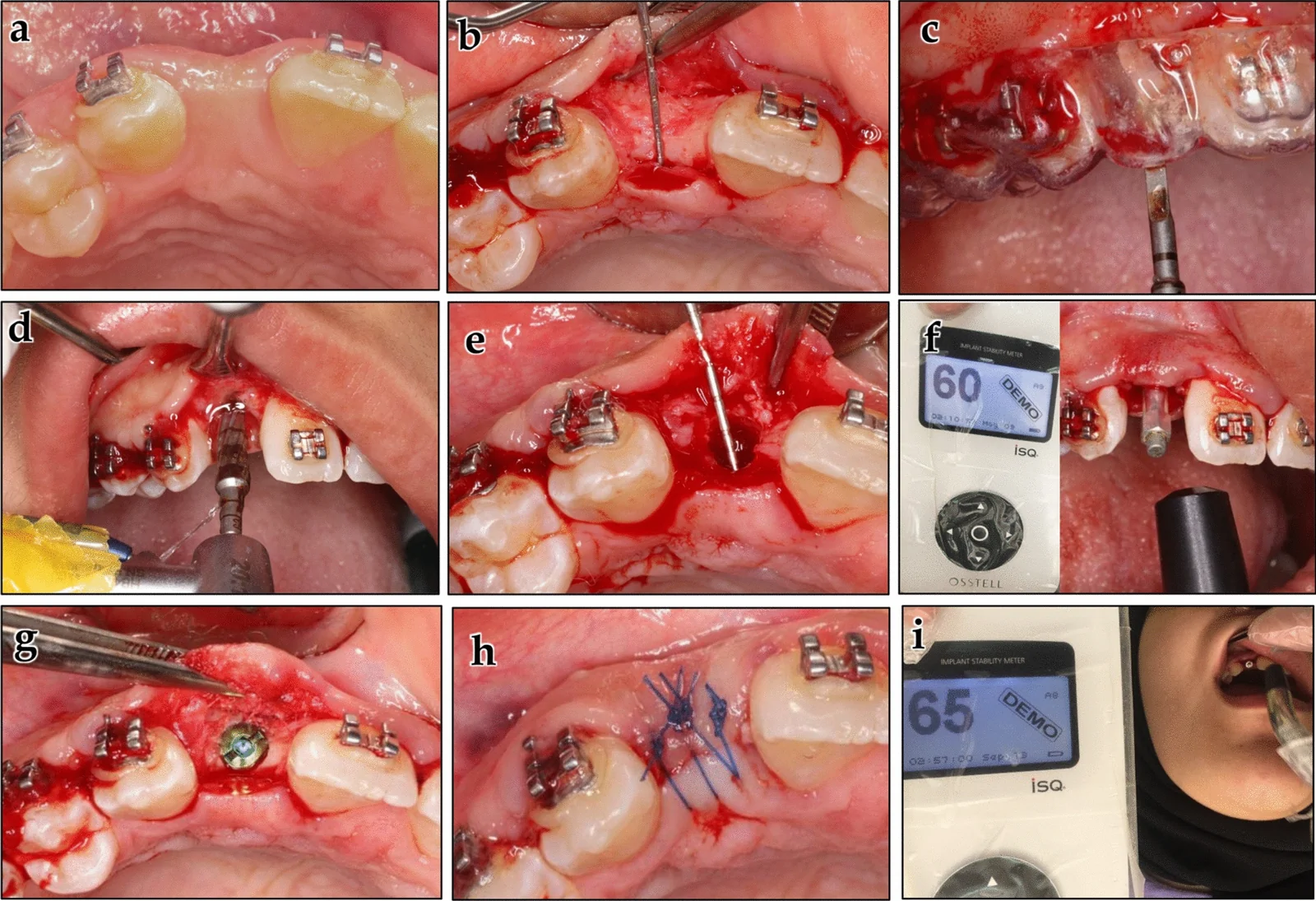
Clinical photographs showing **a** Preoperative occlusal view in Densah group. **b** Measurement of alveolar ridge width after full-thickness muco-periosteal flap reflection. **c** Conventional acrylic surgical stent in place using pilot drill in clockwise rotation. **d** Densah burs used counterclockwise (in the osteotomy site). **e** Measurement of osteotomy site. **f** Using Osstell to measure implant stability immediately after implant placement. **g** Placement of cover screw. **h** Suturing. **i** Using Osstell to measure implant secondary stability 16 weeks postoperatively

**Fig. 2:**
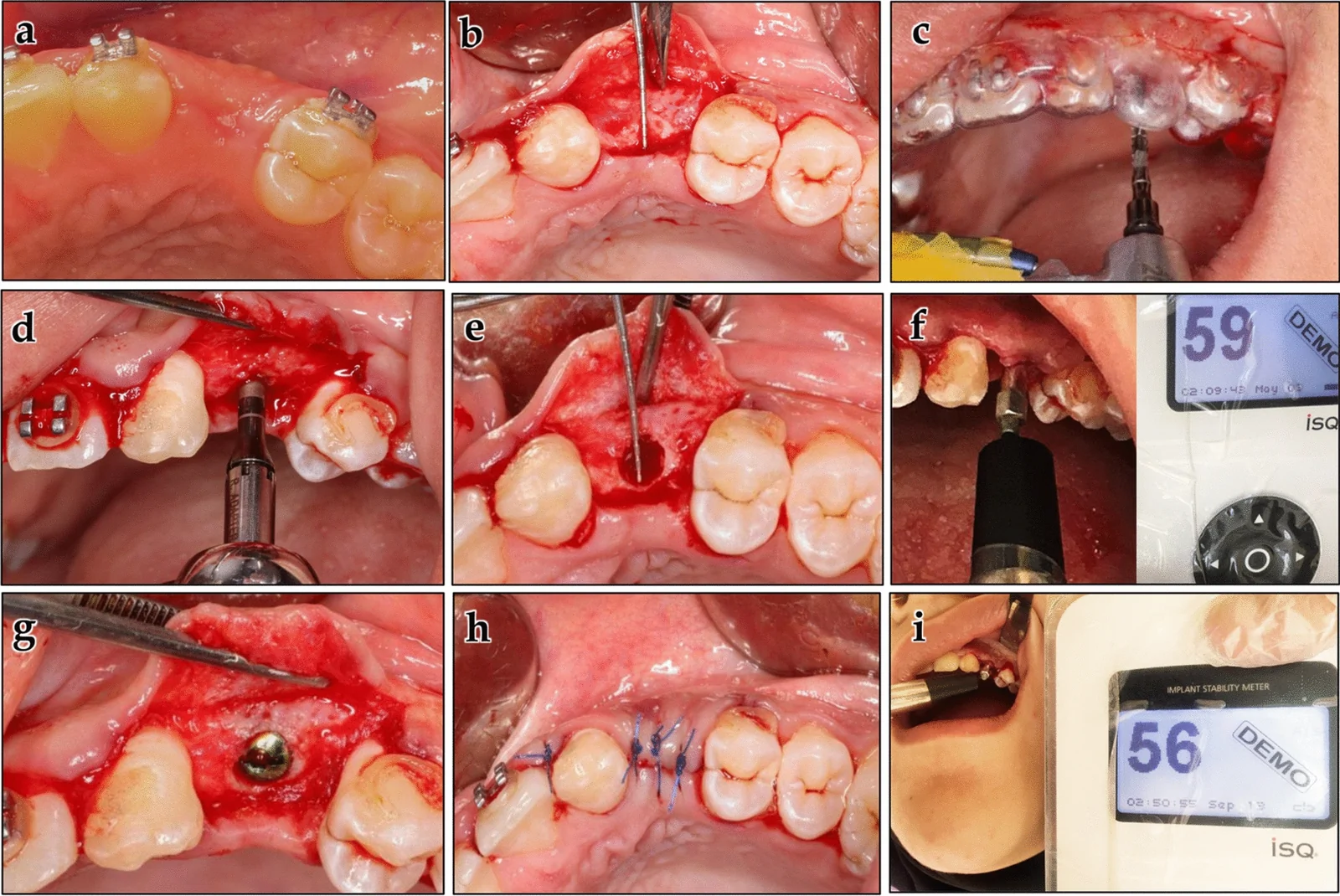
Clinical photographs showing **a** Preoperative occlusal view in conventional manual bone expander group. **b** Measurement of alveolar ridge width after full-thickness muco-periosteal flap reflection. **c** Conventional acrylic surgical stent in place using pilot drill in clockwise rotation. **d** Microdent conventional manual bone expander used in clockwise direction in the osteotomy site using ratchet. **e** Measurement of osteotomy site. **f** Using Osstell to measure implant stability immediately after implant placement. **g** Placement of cover screw. **h** Suturing. **i** Using Osstell to measure implant secondary stability 16 weeks postoperatively

All surgical procedures were performed by a single experienced periodontist (M.H.), ensuring consistency throughout the study. The surgical phase commenced with the administration of local anesthesia (Alexandricaine 1/100,000, Alexandria Co. for Pharmaceuticals & Chemical Industries, Awayed Alexandria, Egypt) at the operation site. A horizontal mid-crestal incision was made, followed by full-thickness muco-periosteal flap reflection. A fabricated conventional acrylic surgical stent was placed to ensure accurate implant positioning. An initial 1.7 mm pilot osteotomy was created using a high-speed surgical handpiece and motor, rotating in a clockwise direction. The pilot drill was subsequently used as a paralleling pin for alignment accuracy (Figs. 1a–c, 2a–c).

For the study group (Osseodensification with Densah burs, Versah, LLC, United States), a 2 mm Densah bur (VT1525) was utilized in a counterclockwise direction for OD, applying copious irrigation with a bouncing-pumping motion. Following this, a 3 mm final Densah bur (VT2535) was used in a counterclockwise direction to prepare the osteotomy site for a 3.5 mm implant. Osteotomy site measurements were recorded, and the implant (Dentium USA, United States) was placed (Fig. 1–f).

For the control group (Conventional Bone Expanders, Microdent, Turkey), a 2 mm Microdent expander was inserted into the osteotomy site in a clockwise direction to avoid heat generation. It was left in place for 5–10 s to allow the bone to relax. The expander was then reversed and removed. A 3 mm Microdent expander was used to prepare the osteotomy site for a 3.5 mm implant (Dentium USA, United States), which was immediately placed to prevent bone recoil (Fig. 2d–f).

In both groups, primary implant stability was assessed immediately after placement using an Osstell device and its SmartPeg (Osstell^®^, Integration Diagnostics AB, Göteborg, Sweden). This device employs a piezoelectric transducer to determine the implant stability quotient (ISQ). Finally, a cover screw was placed, and the surgical flap was repositioned and sutured (Figs. 1f–h, 2f–h).

Postoperatively, patients from both groups were prescribed a nonsteroidal anti-inflammatory drug, Diclofenac Potassium (Catafast^®^ 50 mg sachets, Novartis, Ireland), to be taken twice daily for 5 days. Additionally, patients were instructed and motivated to maintain proper oral hygiene. They were advised to begin rinsing with a 0.12% chlorhexidine gluconate mouthwash (Hexitol, Arab Drug Company, Egypt) twice daily, starting on the second postoperative day and continuing for 1 week. Finally, sutures were removed 1 week after surgery.

### Follow-up and data collection

Clinical parameters included the implant stability quotient (ISQ), measured for all implants to assess primary stability immediately after implant insertion using an Osstell^®^. A second ISQ measurement was taken 16 weeks postoperatively during the second surgical phase to evaluate secondary implant stability (Figs. 1i, 2i).

Radiographic parameters were assessed using CBCT scans performed at three time points: preoperatively, immediately postoperatively, and 16 weeks postoperatively. These assessments included measuring ridge expansion (bone width), marginal bone loss, and bone density around the implants in Hounsfield units (HU) using BlueSky Bio^®^ software.

**Fig. 3:**
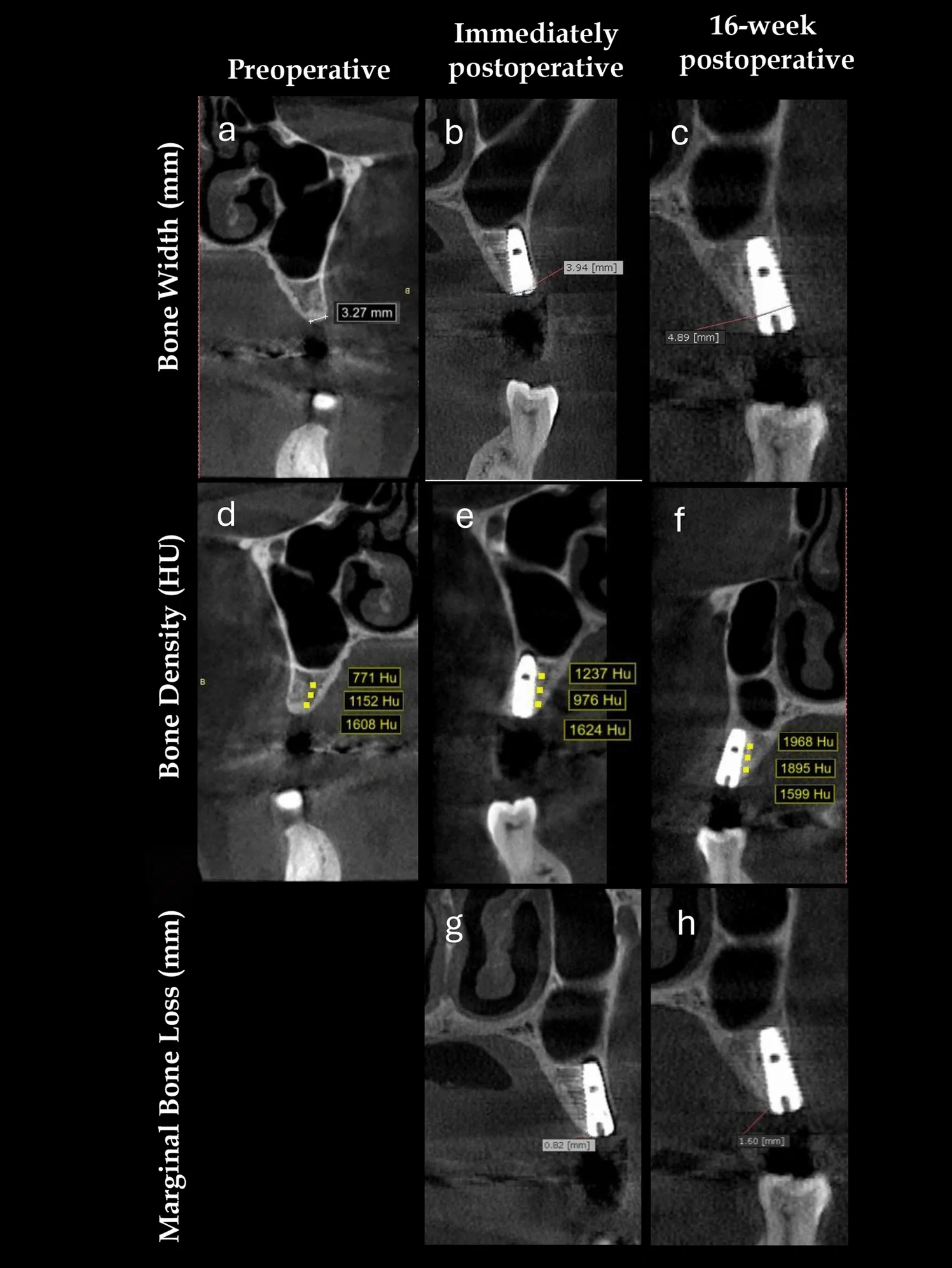
CBCT analysis showing** a** Crestal alveolar ridge width preoperatively. **b** Crestal alveolar ridge width immediate postoperatively. **c** Crestal alveolar ridge width at 16-week follow-up. **d** Three levels taken as a reference to measure average bone density in HU preoperatively. **e** Three levels taken as a reference to measure average bone density in HU immediate postoperatively. **f** Three levels taken as a reference to measure average bone density in HU at 16-week follow-up. **g** Marginal bone level from the implant platform to the most coronal level of bone-to-implant contact at palatal site immediate postoperatively. **h** Marginal bone level from the implant platform to the most coronal level of bone-to-implant contact at palatal site at 16-week follow-up

A. Bone width measurements [35]:

The bucco-lingual width of the bone was measured using the software’s ruler tool at the most coronal part of the ridge crest, extending from the buccal to the lingual border of the bone as shown in Fig. 3a–c.

B. Bone density measurements [36]:

Relative bone density was assessed using the Density Value Measure tool of the BlueSky Plan software (version 4.9.4 by Blue Sky Bio^®^) at three distinct palatal points: the crest of the ridge, 3 mm apical to the crest, and 6 mm apical to the crest as shown in Fig. 3d–f. The mean of these measurements was then recorded.

C. Marginal Bone Loss assessment [37]:

Marginal bone loss was evaluated by measuring the vertical distance from the implant platform (the horizontal interface between the implant and the abutment) to the most coronal level of bone-to-implant contact at palatal sites, both immediately and 16 weeks postoperatively. The difference between these measurements indicated the marginal bone loss (Fig. 3g, h).

The selection of a 16-week follow-up period is grounded in the osseointegration process. Research indicates that implant stability in immediate placement protocols aligns with that of implants placed in native bone after 12 to 16 weeks [38]. This timeframe allows for sufficient bone healing and soft tissue adaptation, providing a reliable assessment of implant integration and success.

All measurements were recorded by a blinded pre-calibrated clinician [39, 40]. Before the study began, two examiners underwent a calibration process. Inter- and intra-examiner reliability were assessed, yielding kappa values between 0.82 and 0.88, indicating excellent agreement both between examiners and over time [41].

### Statistical analysis

The data were entered into the computer and analyzed using IBM SPSS Statistics software, version 25.0 (Armonk, NY: IBM Corp). Shapiro–Wilk test of normality was used to assess the distribution of the quantitative variables. Most of the continuous variables were not normally distributed, so the non-parametric statistics were adopted. Comparisons were carried out between the two studied related not-normally distributed subgroups using the Wilcoxon Signed-Rank test. Mann–Whitney *U* test was used to compare two independent groups for quantitative variables that were not normally distributed. Friedman test was applied to assess differences across more than two related periods for non-normally distributed quantitative variables, followed by Dunn’s post hoc test for pairwise comparisons. Statistical significance was determined at a 5% level.

## Results

After screening 25 potentially eligible patients, 18 individuals aged from 25 to 45 years (6 males and 12 females) were included in the study. All participants met the eligibility criteria and underwent baseline evaluations before being randomly assigned to two study groups, as detailed in Table 1. Each group consisted of nine patients, all presenting with a missing maxillary anterior tooth with a moderate horizontal ridge defect. This defect was defined as an alveolar ridge width of 3–4 mm and a minimum height of 10 mm. The 16-week clinical trial was completed successfully by all participants. Notably, no oral health problems or complications were reported by any patient during the study.

**Table 1:** Comparison between the two studied groups according to demographic data

Demographic data	Group		Test of significance *p* value
	Densah (*n* = 9)	Expander (*n* = 9)	
Age			
*N*	9	9	
Min–Max	29.00–45.00	25.00–42.00	
Mean ± std. deviation	36.56 ± 5.39	35.11 ± 5.23	
SEM	1.80	1.74	*Z*_(MW)_ = 0.536
95% CI for mean	32.41–40.70	31.09–39.13	*p* = 0.592 NS
Median	37.00	35.00	
95% CI for median	32.00–43.00	35.00–42.00	
25th Percentile–75th percentile	32.00–39.00	35.00–38.00	
Sex			
Male (*n* = 6) (33.33%)			
*n* (%)	2 (22.22%)	4 (44.44%)	*χ*^2^_(*df*=1)_ = 1.000
Female (*n* = 12) (66.67%)			*p*_(FE)_ = 0.620 NS
*n* (%)	7 (77.78%)	5 (55.55%)	

*n* number of patients, *Min–Max* minimum–maximum, *SD* standard deviation, *SEM* standard error of mean, *CI* 95% confidence interval, *Z* Z test of Mann–Whitney *U* test, *MW* Mann–Whitney *U*, *χ*^*2*^ Pearson chi-square, *FE* Fisher’s exact test, *df* degree of freedom, *NS* statistically not significant (*p* ≥ 0.05)

**Fig. 4:**
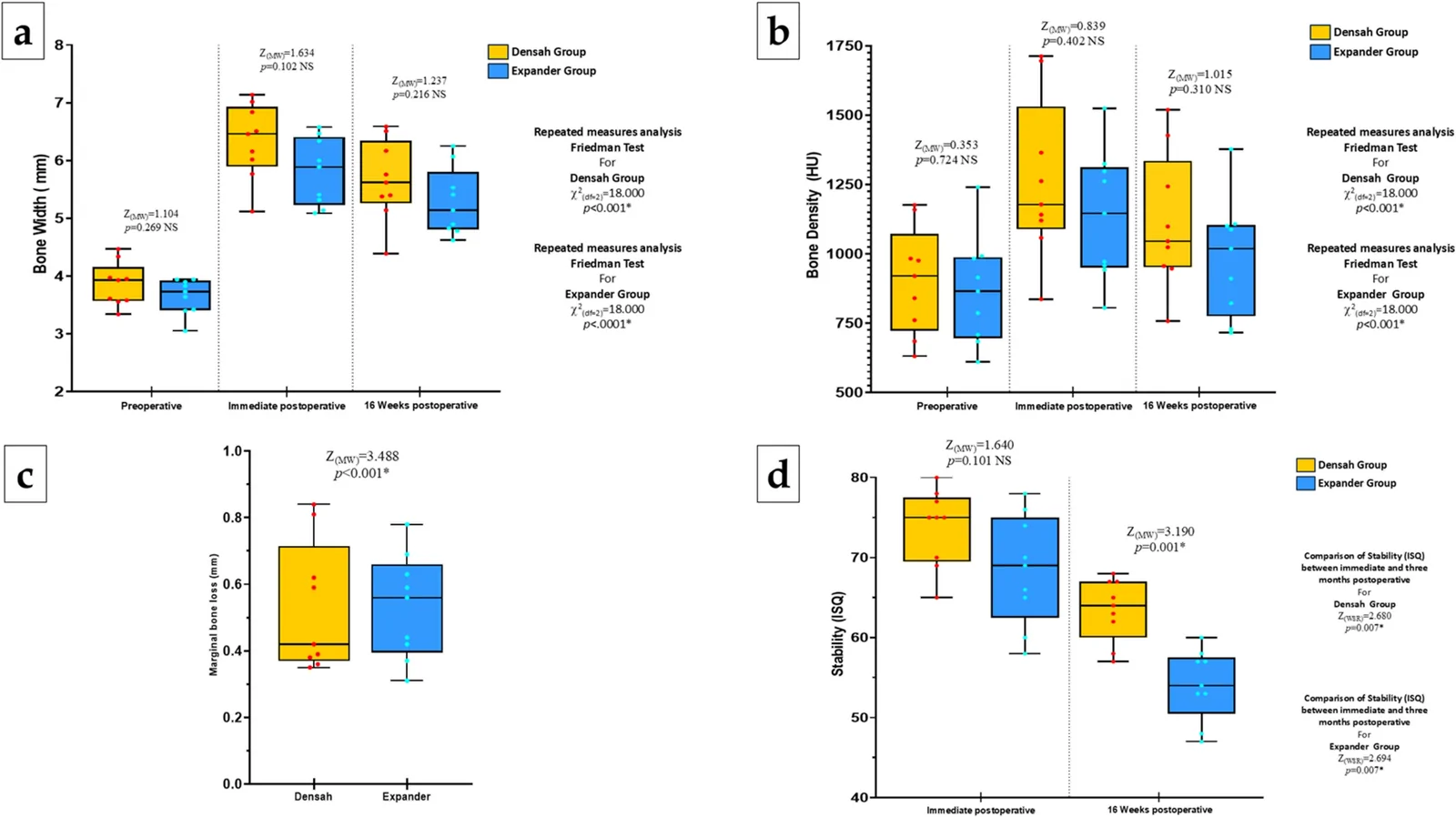
Stagger box and whisker graph of **a** bone width (mm), **b** bone density (HU), **c** marginal bone loss (mm), **d** stability (ISQ) in the studied groups, the thick line in the middle of the box represents the median, the box represents the inter-quartile range (from 25 to 75th percentiles), the whiskers represent the minimum and maximum. The dots represent Individual data points

Concerning bone width, there was no statistically significant difference between the two groups at baseline (*p* = 0.269, 95% CI of difference of medians [− 1.050 to 0.320]), allowing for an unbiased comparison. Similarly, no significant differences were observed immediately postoperatively (*p* = 0.102, 95% CI of difference of medians [− 1.140 to 0.120]) or 16 weeks postoperatively (*p* = 0.216, 95% CI of difference of medians [− 1.030 to 0.310]). However, a highly significant increase in alveolar ridge width was observed within each group individually over the study period (*p* < 0.001) (Table 2; Fig. 4a).

**Table 2:** Comparison of the bone width (mm) in the two studied groups

Bone width (mm)	Group		Test of significance *p* value
	Densah (*n* = 9)	Expander (*n* = 9)	
Preoperative			
Min.–Max	3.34–4.47	3.05–3.94	
Mean ± SD	3.86 ± 0.38	3.65 ± 0.31	*Z*_(MW)_ = 1.104
SEM	0.13	0.10	*p* = 0.269 NS
95% CI for mean	3.57–4.15	3.42–3.88	
Median	3.93	3.73	
95% CI for median	3.58–4.34	3.42–3.94	
25th percentile–75th percentile	3.58–3.97	3.42–3.90	
Immediate postoperative			
Min.–Max	5.12–7.14	5.09–6.58	
Mean ± SD	6.34 ± 0.65	5.80 ± 0.58	*Z*_(MW)_ = 1.634
SEM	0.22	0.19	*p* = 0.102 NS
95% CI for mean	5.84–6.83	5.36–6.25	
Median	6.46	5.89	
95% CI for median	6.02–7.02	5.32–6.47	
25th percentile–75th percentile	6.02–6.84	5.32–6.34	
16 weeks postoperative			
Min.–Max	4.39–6.59	4.62–6.25	
Mean ± SD	5.66 ± 0.70	5.28 ± 0.58	*Z*_(MW)_ = 1.237
SEM	0.23	0.19	*p* = 0.216 NS
95% CI for mean	5.13–6.20	4.84–5.73	
Median	5.62	5.14	
95% CI for median	5.38–6.51	4.83–6.07	
25th percentile–75th percentile	5.38–6.17	4.83–5.53	
Friedman test of significance	*χ*^2^_(df=2)_ = 18.00	*χ*^2^_(df=2)_ = 18.00	
*p*-value	*p* < 0.001	*p* < 0.001	

*n* number of patients, *Min–Max* minimum–maximum, ***SD*** standard deviation, *SEM* standard error of mean, *CI* 95% Confidence interval, *Z* *Z* test of Mann–Whitney *U* test, *MW* Mann–Whitney *U*, *χ*^*2*^ Pearson chi-square, *df* degree of freedom, *NS* statistically not significant (*p* ≥ 0.05)

*Statistically significant (*p* < 0.05)

On the same hand, bone density measurements showed no statistically significant differences between the Densah and Expander groups at any time point: preoperatively (*p* = 0.724, 95% CI of difference of medians [− 393.00 to 146.33]), immediately postoperatively (*p* = 0.402, 95% CI of difference of medians [− 339.00 to 83.50]), or 16 weeks postoperatively (*p* = 0.310, 95% CI of difference of medians [− 27.578 to 8.914]).). However, both groups exhibited notable increases in bone density postoperatively compared to baseline within each group (*p* < 0.001) (Table 3; Fig. 4b).

**Table 3:** Comparison of the bone density (HU) in the two studied groups

Bone density (HU)	Group		Test of significance *p* value
	Densah (*n* = 9)	Expander (*n* = 9)	
Preoperative			
Min.–Max	631.00–1176.00	610.33–1240.33	
Mean ± SD	903.41 ± 192.90	865.02 ± 194.14	*Z*_(MW)_ = 0.353
SEM	64.30	64.71	*p* = 0.724 NS
95% CI for mean	755.13–1051.68	715.79–1014.25	
Median	920.00	865.53	
95% CI for median	761.00–1159.00	708.33–992.00	
25th percentile–75th percentile	761.00–982.67	708.33–982.67	
Immediate postoperative			
Min.–Max	836.33–1712.33	805.33–1524.67	
Mean ± SD	1263.19 ± 288.57	1136.90 ± 233.01	*Z*_(MW)_ = 0.839
SEM	96.19	77.67	*p* = 0.402 NS
95% CI for mean	1041.37–1485.00	957.79–1316.00	
Median	1178.00	1145.90	
95% CI for median	1120.33–1695.67	957.33–1324.33	
25th percentile–75th percentile	1120.33–1365.00	957.33–1298.00	
16 weeks postoperative			
Min.–Max	757.33–1519.00	715.50–1376.93	
Mean ± SD	1113.02 ± 242.83	985.37 ± 213.30	*Z*_(MW)_ = 1.015
SEM	80.94	71.10	*p* = 0.310 NS
95% CI for mean	926.36–1299.68	821.41–1149.33	
Median	1045.33	1018.50	
95% CI for median	956.00–1427.00	822.00–1107.50	
25th percentile–75th percentile	956.00–1243.00	822.00–1100.33	
Friedman test of significance	*χ*^2^_(df=2)_ = 18.00	*χ*^2^_(df=2)_ = 18.00	
*p* value	*p* < 0.001	*p* < 0.001	

*n* number of patients, *Min–Max* minimum–maximum, *SD* standard deviation, *SEM* standard error of mean, *CI* 95% confidence interval, *Z* *Z* test of Mann–Whitney *U* test, *MW* Mann–Whitney *U*, *χ*^*2*^ Pearson chi-square, *df* degree of freedom, *NS* statistically not significant (*p* ≥ 0.05)

*Statistically significant (*p* < 0.05)

Marginal bone loss was comparable between the Densah and Expander groups, with no statistically significant difference (*p* = 0.825, 95% CI of difference of medians [− 0.180 to 0.210]). The median marginal bone loss was 0.42 mm in the Densah group and 0.56 mm in the Expander group, indicating comparable preservation of the crestal bone level around the implants (Table 4; Fig. 4c).

**Table 4:** Comparison of the marginal bone loss (mm) in the two studied groups

Marginal bone loss (mm)	Group	
	Densah group (*n* = 9)	Expander group (*n* = 9)
Min–Max	0.35–0.84	0.31–0.78
Mean ± std. deviation	0.53 ± 0.19	0.53 ± 0.16
SEM	0.06	0.05
95% CI for mean	0.38–0.68	0.41–0.65
Median	0.42	0.56
95% CI for median	0.38–0.81	0.42–0.69
25th percentile–75th percentile	0.38–0.62	0.42–0.63
Test of significance	*Ζ*_(MW)_ = 0.221	
*p*-value	*p* = 0.825 NS	

*n* number of patients, *Min–Max* minimum–maximum, *SD* standard deviation, *SEM* standard error of mean, *CI* 95% confidence interval, *Z* *Z* test of Mann–Whitney *U* test, *MW* Mann–Whitney *U*, *NS* statistically not significant (*p* ≥ 0.05)

In terms of implant stability, it was comparable between the Densah and Expander groups immediately after implant placement (*p* = 0.101, 95% CI of difference of medians [− 11.00 to 1.00]). At 16 weeks, however, the Densah group exhibited significantly higher secondary stability values compared with the Expander group (*p* = 0.001, 95% CI of difference of medians [− 14.00 to − 5.00]). Both groups showed a statistically significant intra-group reduction in stability over time (*p* = 0.007), but the percentage decrease in stability was smaller in the Densah group (− 13.33%) compared to the Expander group (− 20.00%), which was statistically significant (*p* < 0.001, 95% CI of difference of medians [− 9.744 to − 4.058]) (Table 5; Fig. 4d).

**Table 5:** Comparison of the stability (ISQ) in the two studied groups

Stability	Group		Test of significance *P*
	Densah group (*n* = 9)	Expander group (*n* = 9)	
Immediately postoperative			
Min.–Max	65.00–80.00	58.00–78.00	
Mean ± SD	73.78 ± 4.82	68.44 ± 6.89	*Ζ*_(MW)_ = 1.640
Median	75.00	69.00	*p* = 0.101 NS
95% CI of the median	75.00–80.00	65.00–76.00	
25th percentile–25th percentile	70.00–77.00	65.00–74.00	
16 weeks postoperative			
Min.–Max	57.00–68.00	47.00–60.00	
Mean ± SD	63.44 ± 3.91	54.11 ± 4.43	*Ζ*_(MW)_ = 3.190
Median	64.00	54.00	*p* = 0.001*
95% CI of the median	62.00–67.00	53.00–58.00	
25th percentile–25th percentile	62.00–67.00	53.00–57.00	
Test of significance	*Ζ*_(WSR)_ = 2.680	*Ζ*_(WSR)_ = 2.694	
*p* value	*p* = 0.007*	*p* = 0.007*	
Percentage change (%)			
Min.–Max	− 17.95 to − 10.67	− 24.29 to − 17.39	
Mean ± SD	− 13.96 ± 2.41	− 20.78 ± 2.70	*Ζ*_(MW)_ = 3.488
Median	− 13.33	− 20.00	*p* < 0.001*
95% CI of the median	− 15.94 to − 11.43	− 23.08 to − 18.18	
25th percentile–25th percentile	− 15.94 to − 12.31	− 23.08 to − 18.46	

*n* number of patients, *Min–Max* minimum–maximum, *SD* standard deviation, *CI* 95% confidence interval, *Z* *Z* test of Mann–Whitney *U* test, *MW* Mann–Whitney *U*, *WSR* Wilcoxon signed-rank test, *NS* statistically not significant (*p* ≥ 0.05)

*Statistically significant (*p* < 0.05)

## Discussion

Significant alveolar bone resorption presents challenges in dental implant placement, leading to the development of various surgical techniques. Common approaches include autogenous inlays and onlay blocks with or without xenograft particles [42], guided bone regeneration [43], ridge-splitting [44], and distraction osteogenesis [45]. These methods often rely on bone grafts, membranes, titanium meshes, and screws, increasing procedural costs and necessitating a recovery period of 6–9 months before implant placement. Complications, such as dehiscence, infection, donor site morbidity, and graft rejection or failure, are frequent concerns, adding to the complexity of these interventions [46].

Conservative alternatives, such as osteotomes and chisels, aim to enhance bone density and restore the atrophied ridge. However, these techniques are labor-intensive, especially in areas with dense cortical bone. The inability to fully control the direction and amplitude of applied forces may increase patient stress and raise the possibility of minor head injury [47].

The use of thread formers and manual screw expanders represents a less traumatic approach to bone augmentation. These devices gradually expand cortical bone and condense cancellous bone, enhancing implant stability while remaining cost-effective and reliable. This conservative strategy minimizes procedural complexity and reduces patient recovery time, making it a practical alternative in many cases [18, 48].

Osseodensification has emerged as an innovative, non-excavating alternative technique for implant site preparation with expansion and bone condensation. Using motorized high-speed rotary expanders, this method densifies bone along the entire length of the osteotomy while compacting it through autografting. By maintaining bone integrity during osteotomy preparation, OD optimizes bone-to-implant contact, enhances primary stability, and promotes faster healing. This technique directly improves implant success rates by maximizing the quantity and quality of surrounding bone, representing a significant advancement in modern dental implantology [19–21].

In the present study, we evaluated the effectiveness of OD compared to conventional manual bone expanders in enhancing implant stability and achieving ridge expansion across 18 implant sites, equally divided into two groups. The selection criteria for implant sites included an alveolar ridge width of 3–4 mm, with a minimum of 2 mm of cancellous bone and 1 mm of cortical bone on both the buccal and palatal sides. This parameter was chosen to ensure adequate soft tissue support, reduce the risk of buccal bone wall resorption due to compromised blood supply, and minimize the incidence of soft tissue recession around the implants. Furthermore, maintaining the integrity of the bone plate without cracks was crucial to facilitate the effective use of both expanders and Densah burs [49].

Osseodensification in this study employed a counterclockwise drilling direction with speeds ranging from 800 to 1500 rpm, following the guidelines established by Huwais and Meyer [50]. The incorporation of irrigation fluid during osteotomy created a hydrodynamic wave, leading to bone expansion through plastic deformation. The tapered design of the Densah bur contributed to greater crestal expansion compared to the apical region. This design, combined with its atraumatic osteotomy preparation, allowed the inner osteotomy walls to recover, increasing bone-to-implant contact and enhancing biomechanical stability [51]. The findings aligned with Frizzera et al. [52], emphasizing the role of OD in improving ridge thickness and reducing peri-implant defects when paired with tapered implant designs.

In the present study, bone expanders were selected for their non-invasive nature, offering an alternative to guided bone regeneration for ridge widening to facilitate implant insertion. This technique allows for the gradual expansion of a narrow ridge while enabling simultaneous implant placement during the expansion process. The expanders apply controlled force, initially adjusted manually using finger pressure and later with a ratchet mechanism. Each increment of tightening is limited to a half turn, followed by a 30-s waiting period between each turn. This protocol ensures gradual bone expansion, minimizing the risk of placing undue stress on the bone [48].

While the use of expanders typically eliminates the need for bone grafting, there remains a risk of fracture or fissuring of the buccal bone plate during the expansion procedure, which may compromise implant stability [48].

The implants in the present study were positioned in the maxillary anterior region, characterized by soft, low-density bone, where implant success strongly depends on the quality of the surrounding bone. The maxilla is typically composed of D3 or D4 bone types, with a relatively low bone percentage value. Enhancing the density of bone tissues in contact with the implant surface is critical for achieving optimal primary stability [53]. This enhancement can be accomplished through surgical modifications, such as the use of the OD technique. The OD technique employs a non-subtractive, multi-step drilling protocol using burs with negative rake angles (non-cutting edges), which effectively increase bone density [54].

Studies indicate that the Densah bur facilitates the placement of wider implants in narrow ridges, potentially increasing bone volume by up to 30% [50]. The present study found a significant postoperative increase in bone width in both groups compared to preoperative measurements, with no statistically significant differences between them at any time point. This suggests that both OD and expander techniques are effective in enhancing ridge dimensions. While bone width increased immediately postoperatively in both groups, a slight decrease was observed over 16 weeks, indicating some degree of postoperative remodeling. These findings partially align with those of Mahmoud Riad et al. [55], who reported higher mean bone width values in the Densah bur group compared to the Expander group, though the differences were not statistically significant.

No statistically significant difference in bone density was observed between the groups at any time point. However, the Densah group consistently showed higher values, indicating the OD effect. This increase in peri-implant bone density is likely due to trabecular bone compression induced by OD, which enhances bone density both immediately after surgery and overtime. The Densah drills improve bone density around the implant through a compaction-autografting mechanism, consolidating rather than removing bone at the osteotomy site. This process strengthens the interlocking between bone and implant, facilitating rapid osseointegration [56]. These findings align with Ahmed et al. [57], who reported a significant increase in peri-implant bone density in the OD group immediately postoperatively and at 6 months.

Our study found no significant difference between the Densah and Expander groups in primary implant stability, but at 16 weeks, the Densah group showed significantly higher values, with no significant marginal bone loss observed in either group. This improved stability in the Densah group is likely due to osseointegration, where OD enhances bone mineral density around the implant, improving secondary implant stability [50].

In line with Arafat and A Elbaz [58], who found no immediate postoperative difference in implant stability between osteotome and OD techniques, but noted significant improvement at 16 weeks in the Densah group, our study also found enhanced secondary stability over time with Densah burs compared to the Expander Group. Similarly, Sultana et al. [59] reported slightly higher values with Densah burs but no statistically significant difference compared to conventional drills. In contrast, Kothayer and Abdelfattaha [60] found that Densah bur drilling significantly increased primary implant stability compared to traditional drilling, while our study highlighted the positive effect of OD on secondary stability over time.

However, our study observed a decline in stability at 16 weeks compared to immediately after surgery. This decline is likely due to the trabecular microfractures caused by OD, which induce high strains at the bone-implant interface, prolonging the resorption period and delaying secondary stability [61]. Trabecular fractures require extensive remodeling time, further postponing implant stability. Additionally, the remodeling of pre-existing bone and the time needed for new bone formation on the implant surface may contribute to the delayed establishment of secondary stability [61].

In contrast to our findings, a study by Abdel-Rahman et al. [62], examined the potential divergent outcomes between OD and expander techniques. The findings indicated no difference in implant stability between the two groups at various time intervals (implant placement, loading time, and 6, 12, and 36 months post-loading). Conversely, our study demonstrated that the Densah group exhibited statistically significantly higher stability than the Expander group 16 weeks postoperatively, although no statistically significant difference was observed immediately postoperatively. This discrepancy may be attributed to the smaller sample size, the inclusion of only females with a higher mean age, different implant sites (molar and premolar), and variations in alveolar width and height compared to our study.

The approach of increasing bone density around implants to enhance primary stability has been extensively explored, particularly in anatomically restricted regions where achieving adequate initial implant stability is challenging [61]. The bone expansion and compaction achieved through the OD technique, without observed complications, facilitated simultaneous implant placement with high levels of both primary and secondary stability in narrow alveolar ridges of low bone quality. Previous studies have reported comparable outcomes when OD was used to enhance implant stability, demonstrating improved bone condensation and ridge expansion compared with traditional techniques. These findings align with the present results, which demonstrated that both techniques achieved significant intra-group increases in bone width and density, yet OD yielded superior secondary stability values. Collectively, these outcomes highlight the clinical advantages of OD in optimizing the bone–implant interface and improving mechanical stability.

The present findings further confirm that OD significantly enhances secondary implant stability compared with manual bone expanders. This outcome is supported by the densification-induced increase in peri-implant bone density and improved bone–implant contact. Nevertheless, the biological mechanisms underlying these improvements, including trabecular compaction and the autografting of bone particles generated during densification, remain partially speculative and warrant further histological and biomechanical investigation [22, 63].

Despite these encouraging outcomes, several limitations should be acknowledged. One limitation is the use of a convenience sampling technique; however, this method is commonly employed in clinical trials, where examiners screen and recruit eligible participants based on accessibility [64, 65]. Additionally, the relatively small sample size may have affected the statistical power of the study. Therefore, further research with larger sample sizes is warranted to enhance the validity and generalizability of the findings. Another key limitation of this study is the relatively short follow-up period. Longer follow-up durations are recommended in future research to evaluate long-term implant success. Furthermore, while OD demonstrated promising results in healed ridges, additional research is needed to evaluate its performance in immediate implant placement protocols. Finally, the retrospective nature of the clinical trial registration constitutes a limitation in terms of methodological transparency although all ethical approvals and study procedures were conducted in accordance with institutional and international standards.

## Conclusion

Within the limitations of this study, Densah burs have proven to be an effective clinical tool for ridge expansion, enabling successful implant placement with adequate primary stability and enhanced secondary stability. They offer a less-invasive, cost-effective, and more straightforward approach to bucco-lingual alveolar ridge expansion compared to conventional manual expanders, particularly in cases where the alveolar bone width is less than 3 mm, making the conventional ridge-splitting technique unsuitable. Additionally, OD reduces the incidence of buccal peri-implant defects, facilitating simultaneous implant placement and bone grafting. These findings suggest that OD is a promising alternative to conventional drilling techniques, especially in challenging clinical scenarios where bone volume is limited, and esthetic outcomes are crucial. While the short-term outcomes are promising, longer follow-up periods are required before drawing up conclusions regarding early loading or long-term success rates.

## Data Availability

All data included in this current study are available from the corresponding author upon request.
